# Next Generation Sequencing and Genetic Analyses Reveal Factors Driving Evolution of Sweetpotato Viruses in Uganda

**DOI:** 10.3390/pathogens13100833

**Published:** 2024-09-26

**Authors:** Joanne Adero, Godfrey Wokorach, Francesca Stomeo, Nasser Yao, Eunice Machuka, Joyce Njuguna, Denis K. Byarugaba, Jan Kreuze, G. Craig Yencho, Milton A. Otema, Benard Yada, Mercy Kitavi

**Affiliations:** 1 National Crops Resources Research Institute, National Agricultural Research Organization, Kampala P.O. Box 7084, Uganda; miltonotema49@gmail.com (M.A.O.); yadabenard21@gmail.com (B.Y.); 2 Biosciences Eastern and Central Africa, International Livestock Research Institute (BecA-ILRI) Hub, Nairobi P.O. Box 30709, Kenya; stomeofra@gmail.com (F.S.); n.yao@cgiar.org (N.Y.); e.machuka@cgiar.org (E.M.); joyceleyn@gmail.com (J.N.); 3 College of Veterinary Medicine Animal Resources and Biosecurity, Makerere University, Kampala P.O. Box 7062, Uganda; denis.byarugaba@mak.ac.ug; 4 Department of Biology, Faculty of Science, Muni University, Arua P.O. Box 725, Uganda; wokosiki@gmail.com; 5 International Potato Centre, CIP Headquarters Lima, Avenida La Molina 1895, La Molina Apartado Postal 1558, Lima 15024, Peru; j.kreuze@cgiar.org; 6 Department of Horticultural Science, North Carolina State University, 214 Kilgore Hall, P.O. Box 7609, Raleigh, NC 27695, USA; craig.yencho@gmail.com; 7 International Potato Centre, SSA Regional Office, Nairobi P.O. Box 25171, Kenya

**Keywords:** sweet potato viruses, whole genomes, recombination, phylogenetic analysis

## Abstract

Sweetpotato (*Ipomoea batatas* L.) is an essential food crop globally, especially for farmers facing resource limitations. Like other crops, sweetpotato cultivation faces significant production challenges due to viral infections. This study aimed to identify and characterize viruses affecting sweetpotato crops in Uganda, mostly those associated with sweetpotato virus disease (SPVD). Infected leaf samples were collected from farmers’ fields in multiple districts spanning three regions in Uganda. MiSeq, a next-generation sequencing platform, was used to generate reads from the viral nucleic acid. The results revealed nine viruses infecting sweetpotato crops in Uganda, with most plants infected by multiple viral species. Sweet potato pakakuy and sweet potato symptomless virus_1 are reported in Uganda for the first time. Phylogenetic analyses demonstrated that some viruses have evolved to form new phylogroups, likely due to high mutations and recombination, particularly in the coat protein, P1 protein, cylindrical inclusion, and helper component proteinase regions of the potyvirus. The sweet potato virus C carried more codons under positive diversifying selection than the closely related sweet potato feathery mottle virus, particularly in the P1 gene. This study provides valuable insights into the viral species infecting sweetpotato crops, infection severity, and the evolution of sweet potato viruses in Uganda.

## 1. Introduction

Over the past decade, sweetpotato (*Ipomoea batatas* (L.) Lam.) has gained prominence as a major crop, making it the seventh most important food crop consumed worldwide [1]. Sweetpotato is grown mainly by smallholder women farmers and serves as an outstanding crop for food and nutrition security in sub-Saharan Africa (SSA) [2,3]. In Uganda, production has gradually declined over the years, dropping the country from the second to the tenth largest sweetpotato producer in the world [1]. This decline is mainly attributed to pests and diseases [4].

Worldwide, viruses are the leading pathogens in crop diseases, causing yield losses that have a great economic and social impact [5]. However, viruses are largely underappreciated, often due to the subtle nature of their symptoms. Globally, sweetpotato production is constrained by diseases caused by RNA and DNA viruses. Over 35 viruses are known to infect sweetpotato [6] but only seven of these viruses have been reported in Uganda: sweet potato feathery mottle virus (SPFMV), sweet potato virus C (SPVC), sweet potato chlorotic stunt virus (SPCSV), sweet potato chlorotic flecks virus (SPCFV), sweet potato caulimolike virus (SPCaLV), sweet potato mild mottle virus (SPMMV) and sweet potato leaf curl virus (SPLCV) [7,8,9,10,11].

Sweet potato feathery mottle virus (SPFMV; family *Potyviridae*, genus *Potyvirus*) is the most dominant pathogen of cultivated sweetpotato worldwide [12,13]. Previous classification of SPFMV strains into O, EA and RC groups based on geographical location and biological properties has been misleading. This is because the first detection and distribution of SPFMV strains is no longer localized to countries, continents or regions. Similarly, biological properties of the phylogenetic group SPFMV RC now contain isolate names that do not cause ‘Russet Crack’ in sweetpotato storage roots. More recently, a new classification proposal has grouped the isolates into phylogroups A, which comprises strain O and strain EA, and Phylogroup B, which comprises strain RC [8,12,14]. Sweet potato virus C (SPVC; family *Potyviridae*, genus *Potyvirus*; species *Potyvirus cebatatae*), formerly called SPFMV strain C, also occurs worldwide and often in co-infections with SPFMV [15,16].

In single infections, SPFMV may present mild or no symptoms in sweetpotato plants. When co-infected with sweet potato chlorotic stunt virus (SPCSV; family *Closteroviridae,* genus *Crinivirus*), SPFMV symptoms become severe, resulting in a sweet potato virus disease (SPVD) syndrome. SPVD is the most economically important disease in sweetpotato, causing yield losses of 70–100% worldwide [7,17]. This is because SPCSV suppresses RNA silencing-mediated viral defense in sweetpotato [18], making it relevant in co-infections. In addition to SPVD, SPCSV also causes synergistic diseases with all other sweetpotato viruses, resulting in synergistic diseases that may severely affect sweetpotato yield [19]. In Uganda, several widespread sweetpotato virus disease complexes have been reported in major sweetpotato growing regions. These complexes have contributed to the elimination of local cultivars and farmer-preferred varieties [7,9]. Despite the widespread incidence of sweetpotato viral disease complexes in East Africa, there have been few studies on their molecular variability based on sequence analysis [20,21].

Compared to most pathogens, viruses are challenging to detect and identify because they are obligate parasites that cannot proliferation outside a host cell and are polyphyletic, lacking a single gene common to all viruses that can be used for general diagnostic tests [22]. Furthermore, the frequent occurrence of mixed viral infections and synergistic complexes, like SPVD, complicates the detection and identification of infecting viruses in sweetpotato [7].

Management of SPVD is indispensable for the sustainable production of sweetpotato. Several control methods used include cultural practices, phytosanitary measures, and the use of clean and virus-free planting materials. Host resistance has been seen as the most effective and sustainable approach for the management of SPVD among small-scale farmers [23]. Breeding for virus-resistant varieties has been a challenge due to the self- and cross-incompatibility and the genetic complexity of the hexaploid sweetpotato genome. Therefore, virus resistance levels found in breeding populations to date occur at very low frequencies and resistance that works against one virus strain may not be effective against another [24]. Nevertheless, host resistance may be broken down by local virus strains, making information on circulating viruses vital for the effective breeding of resistant local varieties. Knowledge of the virus’s genetic diversity not only serves as guidance for management strategies but also helps to understand the virulence, spread and emergence of epidemics [21].

Conventional detection of plant viruses has been accomplished using a combination of molecular, serological and biological indexing, each method having unique advantages. However, these methods necessitate prior knowledge of the virus in question. Next-generation sequencing (NGS) is a hypothesis-free approach to detection that does not require prior information on the virus sequence. Moreover, advances in NGS technologies have allowed for a metagenomic approach to virus detection that has greatly improved plant virus discovery, diagnostics and evolutionary studies and has been helpful for detecting viral co-infections in many plants [25,26].

We utilized the Illumina MiSeq platform to generate virus genome reads for the detection and sequencing of sweetpotato viruses in Uganda. This approach provides insights into their genetic variability and the evolutionary factors at play. This knowledge is crucial for effective sweetpotato virus surveillance and is valuable to national breeding programs in the region, as it enables targeted breeding for virus resistance and enhances integrated disease management.

## 2. Materials and Methods

### 2.1. Sample Collection 

**Figure 1:**
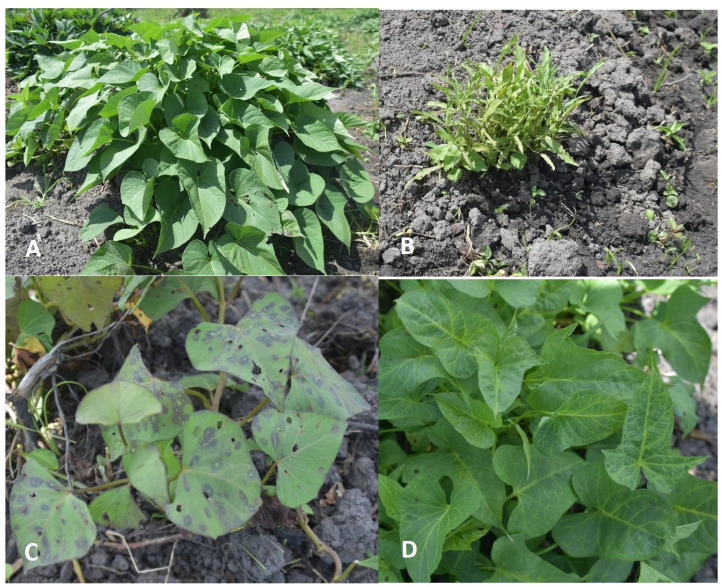
Virus symptoms in sweetpotato plants observed in farmer’s fields: (**A**) healthy sweetpotato crop; (**B**) stunting; (**C**) chlorotic ring spot and purpling; (**D**) chlorosis and vein banding.

Vines collected from a previous cross-sectional survey conducted in 16 districts in Uganda were planted and established in a screen house at the National Crops Resources Research Institute (NaCRRI). The districts were in the following regions: central (Wakiso, Mukono, Mpigi, Luweero, Nakasongola); eastern (Iganga, Serere, Soroti, Kumi, Kamuli, Mbale); and western (Bushenyi, Kasese, Kabarole, Mubende, Hoima). At the time of the survey, each sweetpotato field was scouted for virus-like symptoms. Plants showed symptoms including vein clearing, mottling, yellowing, chlorotic vein flecks, chlorotic spots, leaf distortion, stunting and purpling of leaves, among others [7], as shown in Figure 1.

From the established plants in the screen house at NaCRRI, three representative leaf samples per district were collected for sequencing and analysis. The selection criteria for sampling were based on the level of infection, with mild, moderate and severe virus symptoms, giving a total of 48 samples. Leaf samples were collected using sterile forceps and stored in liquid nitrogen to prevent the degradation of RNA. The samples were then transported to the Biosciences Eastern and Central Africa–International Livestock Research Institute (BecA-ILRI) laboratory in Nairobi, Kenya, for sequencing.

### 2.2. RNA Extraction, Library Preparation and Illumina MiSeq Sequencing

Total RNA was extracted from approximately 0.1 g of infected young leaf samples using an RNeasy Plant Mini Kit (Qiagen, USA), following the manufacturer’s instructions and eluted in 50 µL of RNase-free water. RNA quality was assessed using 1% (*w*/*v*) agarose gel electrophoresis at 100 V for 30 min. The concentration was measured using a high sensitivity RNA assay kit (RNA HS) with a Qubit 2.0 fluorometer (Life Technologies, Carlsbad, CA, USA), as described in the manufacturer’s protocol, and the RNA was then stored at −80 °C. Metagenomic libraries were prepared from 1.0 µg of total RNA using the Illumina TruSeq RNA preparation protocol (Illumina, San Diego, CA, USA) according to the manufacturer’s instructions. Briefly, total RNA was subjected to first-strand cDNA synthesis using SuperScript™ II reverse transcriptase (ThermoFisher Scientific, Waltham, MA, USA), random primers and first-strand master mix. Subsequently, second-strand synthesis was carried out using DNA polymerase, RNase H and the second-strand master mix provided with the kit. Double-stranded cDNA was purified using Agencourt AMPure XP magnetic beads (Beckman Coulter, Indianapolis, IN, USA). End-repair and subsequent adenylation of the 3′ end of the synthesized ds cDNA was performed before ligation of Illumina adaptors. Unique adaptors for each library were ligated to the 5′ and 3′ ends of the ds cDNA, and the libraries were enriched through polymerase chain reaction (PCR) under the following cycling conditions: one cycle of 98 °C for 30 s; 15 cycles of 98 °C for 10 s, 60 °C for 30 s and 72 °C for 30 s; with a final extension at 72 °C for 5 min. The quality of the libraries was confirmed using an Agilent 2200 TapeStation (Agilent Technologies Inc., Santa Clara, CA, USA), and the concentration of the cDNA libraries was estimated with the high sensitivity DNA (dsDNA HS) kit using a Qubit 2.0 fluorometer Life Technologies, Carlsbad, CA, USA). The libraries were normalized and pooled at equal molar concentrations for multiplexing. Paired-end sequencing was carried out using a 2 × 300 cycle PE V3 Illumina kit (Illumina, San Diego, CA, USA) on an Illumina MiSeq sequencer.

### 2.3. Sequence Data Analysis

Sequence reads were assessed for quality using FastQC (FastQC Babraham Bioinformatics, “https://www.bioinformatics.babraham.ac.uk/projects/fastqc/” (accessed on 13 December 2017). Low-quality reads (below 20 Phred scores), sequencing adapters and PCR primers were removed using Trimmomatic v.0.33 [27]. A set of 10 samples of low quality was removed from the workflow, leaving 38 FASTQ files for downstream analysis. The plant host genome was removed by mapping reads to the host representative reference genome, *Ipomoea trifida*, NSP306 v3; “http://sweetpotato.uga.edu/” (accessed on 20 December 2017) [28], using Bowtie2 v.2.2.8 [29]. Reads not mapped to the reference genome were assembled de novo to obtain contigs and scaffolds using SPAdes v.3.10.1 [30]. The assembled scaffolds were checked against the National Center for Biotechnology Information (NCBI) plant virus database [31] using BLASTn and BLASTx [32] online tools to determine either partial or whole genomes based on their length and match (coverage%, identity and E value of query results). Molecular analysis was carried out only on de novo genome sequences that were greater than 97% of the length of the virus genome sequences in NCBI. For further analysis, reference genome sequences with the best alignment descriptions above 90% identity were downloaded from NCBI GenBank, as listed in Supplementary Table S4, uploaded to CLC Genomics Workbench v23.0.8, “https://www.qiagenbioinformatics.com” (accessed on 13 February 2024) along with the obtained de novo genome sequences. Nucleotide alignment, reference mapping and gene annotation of whole de novo genome sequences were performed against NCBI reference genomes in CLC Genomics Workbench. Respective protein sequences from the de novo sequences were extracted.

### 2.4. Nucleotide Sequence Identity and Phylogenetic Analysis

Global identity and similarity statistics of virus sequences were performed on whole genome sequences using the Sequence Demarcation Tool version 1.3 (SDTv1.3) [33] to generate a sequence identity distribution plot and a color-coded similarity matrix. Phylogenetic analysis was conducted for the protein-coding sequences of SPCFV, SPFMV and SPVC using MEGA 11.0.13 [34]. Complete nucleotide sequences and the deduced amino acid sequences of de novo sequences and previously reported virus sequences were aligned using ClustalW methods in Mega 11. Phylogenetic trees were inferred using the maximum likelihood method with the best-fit nucleotide and amino acid substitution models, which was the General Time Reversible model, and bootstrap values were calculated using 100 random replications.

### 2.5. Recombination Analysis

Recombination was examined on whole genome sequences using the recombination detection program (RDP) v.4.95 [35] and recombination events predicted by at least three of packages, RDP [36], GENECONV [37], Bootscan [38], MaxChi [39], Chimaera [40], 3Seq [41] and SiScan [42] in the RDP4 program, with default settings and a Bonferroni *p*-value of 0.05.

### 2.6. Nucleotide Diversity, Genetic Differentiation and Neutrality Tests

To understand the diversification in protein coding regions of sweetpotato potyviruses, nucleotide diversity, the number of segregating sites, the average number of nucleotide differences and the total number of mutations were calculated based on phylogenetic sub-populations using DnaSP v. 5 [43]. Fixation index Fst was used to determine genetic differentiation and gene flow between the different sub-populations observed in the study. Neutrality tests based on Tajima’s [44] and Fu and Li’s parameters [45] were performed to detect natural selection occurring in the different subpopulations.

### 2.7. Detecting Signatures of Selection within Sweetpotato Potyviruses

Selection across amino acid sites across the entire genes of viruses was analyzed using three packages in the Datamonkey Adaptive Evolution Server to detect positive diversifying selection or purifying selection. The Fixed Effects Likelihood (FEL) [46] was employed to infer nonsynonymous (dN) and synonymous (dS) substitution rates per site, detecting both purifying and positive selection. Additionally, the Mixed Effects Model of Evolution (MEME) [47] was used to identify sites under positive diversifying selection. A threshold *p*-value of 0.05 was applied to identify the sites under selection.

## 3. Results

### 3.1. Virus Identification

**Figure 2:**
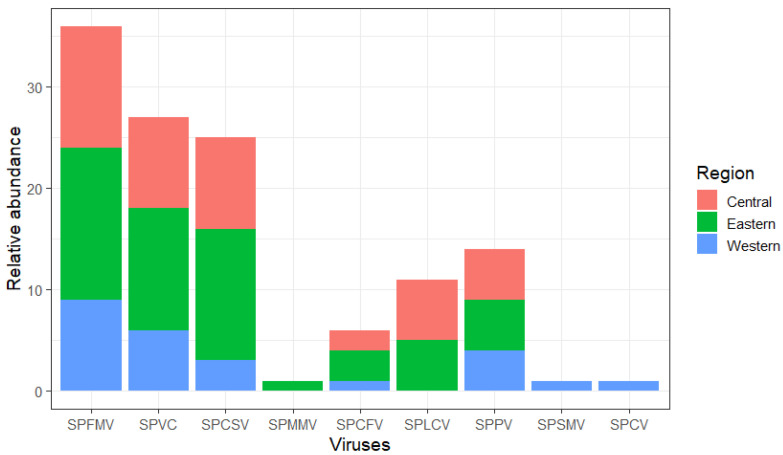
Frequency of different sweet potato viruses detected across survey regions (Central, Eastern and Western). The viruses detected include SPFMV (sweet potato feathery mottle virus), SPVC (sweet potato virus C), SPCSV (sweet potato chlorotic stunt virus), SPMMV (sweet potato mild mottle virus), SPCFV (sweet potato chlorotic fleck virus), SPLCV (sweet potato leaf curl virus), SPPV (sweet potato pakakuy virus), SPSMV_1 (sweet potato symptomless_1 virus) and SPCV (sweet potato collosive virus).

High-throughput sequencing was conducted on 48 samples, of which 38 leaf samples passed the quality check, resulting in a total of 42,604,740 reads before cleaning and 41,441,888 reads after quality trimming and removal of poor-quality reads (Supplementary Table S1), with an average GC content of 49.9%. BLASTn and BLASTx analyses of de novo assemblies, including both partial and whole virus genome sequences, identified five RNA viruses and four DNA viruses (Figure 2; Table 1). The nucleotide sequence identities ranged between 81% and 100%, compared to virus sequences in NCBI GenBank.

The aphid-transmitted RNA viruses SPFMV and SPVC were the most frequently detected and found in 95% and 71% of the samples, respectively. Both SPFMV and SPVC showed high frequencies across all surveyed regions, indicating widespread distribution in sweetpotato. The whitefly-transmitted SPCSV was the third most detected virus and present in 66% of the plants tested. However, SPCSV was less common in districts from the western region compared to the central and eastern regions. SPCFV had a lower detection rate of 16% across all surveyed regions. Another RNA virus, SPMMV, was marginally detected at 3% and found in only one sample collected from the Mbale district (Table 1; Figure 2).

The frequency of occurrence of DNA viruses was low in comparison with RNA viruses. Although this study reports the occurrence of SPPV for the first time in Uganda, it had the highest occurrence of 37% among DNA viruses and was detected in all regions surveyed. This was followed by SPLCV, which was detected in 29% of samples tested and was found in only central and western Uganda. SPCV and SPSMV_1 had the lowest detection rates of 3% and were both detected in western Uganda. This study also reports the first occurrence of SPSMV_1 in Uganda (Table 1; Figure 2).

The frequency of DNA viruses was notably lower compared to RNA viruses. This study reports, for the first time in Uganda, the occurrence of SPPV, which had the highest detection rate among DNA viruses at 37% and was detected across all surveyed regions. Following SPPV, SPLCV was detected in 29% of the samples and was found only in central and western Uganda. SPCV and SPSMV_1 had the lowest detection rates at 3%, and both were found in western Uganda. Additionally, this study marks the first occurrence of SPSMV_1 in Uganda (Table 1; Figure 2).

### 3.2. Co-Infection of the Viruses

Co-infections of sweetpotato with multiple viruses were common, with only three plants showing single infection by SPFMV. Mixed infections involving up to six viruses in one host plant were detected, including co-infections between DNA and RNA viruses. SPFMV, SPVC and SPCSV were the most frequently involved viruses in co-infections across all regions. Notably, co-infections also occurred between SPFMV strain O and SPFMV strain EA in 34% of the tested samples. SPCFV was predominantly detected in the presence of SPFMV, SPVC and SPCSV (Table 1).

Partial virus sequences obtained from this study were discarded, and only whole de novo genome sequences above 97% were used for further molecular analysis.

### 3.3. Phylogenetic Analysis and Nucleotide Sequence Identities

Three de novo whole length genome sequences of SPCFV, eleven de novo whole genome sequences of SPFMV and eight de novo whole genome sequences of SPVC were obtained. These sequences were deposited in NCBI GenBank, and accession numbers are provided (Supplementary Tables S3 and S4).

#### 3.3.1. Phylogenetic Analysis and Nucleotide Sequence Identities of Sweet Potato Chlorotic Fleck Virus

**Figure 3:**
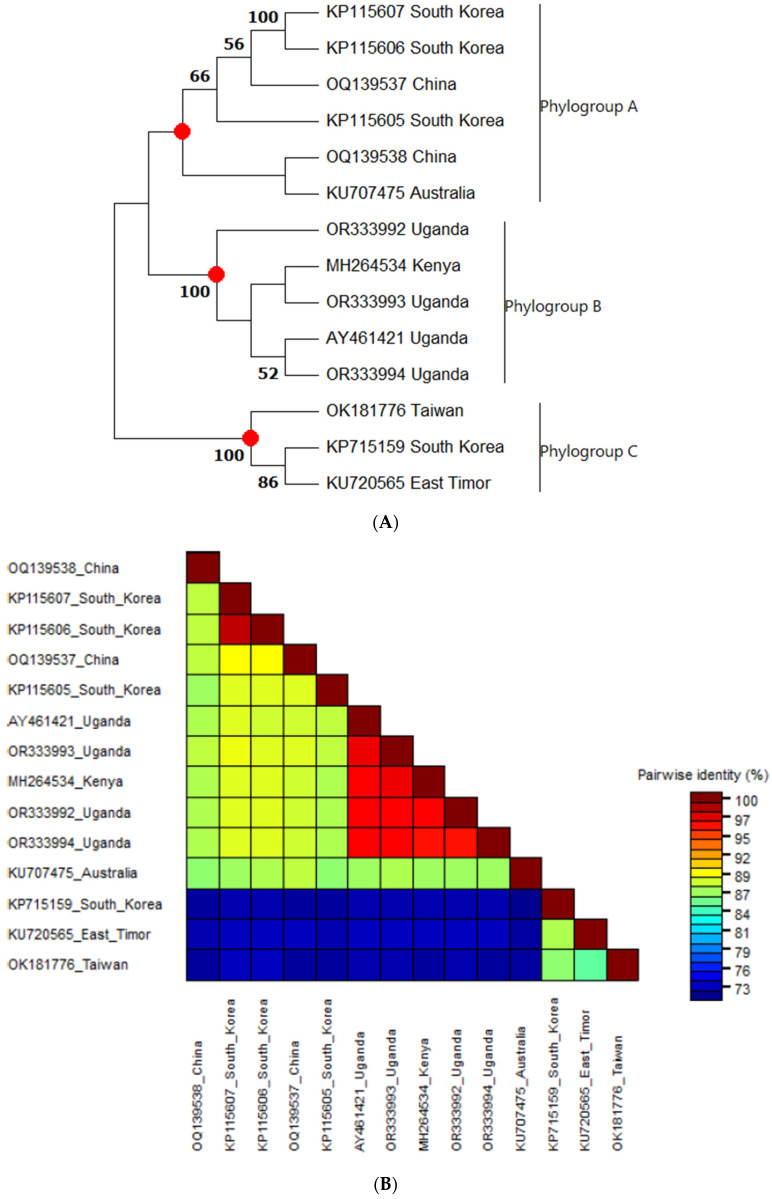
(**A**) Phylogenetic tree constructed using whole genome sequences of sweet potato chlorotic fleck virus. Phylogenetic tree was inferred using the maximum likelihood method based on the General Time Reversible model in MEGA11. Numbers at each node indicate bootstrap values. (**B**) Color-coded nucleotide identity matrix displaying global identity and similarity statistics between SPCFV whole genome sequences.

Phylogenetic analysis of the nucleotide sequences for the protein coding region of SPCFV using maximum-likelihood methods revealed the presence of three major clusters, designated as phylogroups A, B and C (Figure 3). Phylogroup A was predominantly comprised of sequences from Asian countries, such as South Korea, Taiwan and East Timor. Phylogroup B consisted of sequences from East Africa (specifically Uganda and Kenya), while phylogroup C included sequences from Australia and Asia (including China and South Korea) (Figure 3A). The highest nucleotide identity was observed among isolates in phylogroup B (sequences from East Africa), indicating lower diversity compared to phylogroups C and A, which exhibited a relatively lower nucleotide identity. Phylogroup C showed a nucleotide identity below 80% with both phylogroups A and B, indicating a more distant relationship with both phylogroups B and A (Figure 3B).

#### 3.3.2. Phylogenetic Analysis and Nucleotide Sequence Identities of Sweet Potato Feathery Mottle Virus

**Figure 4:**
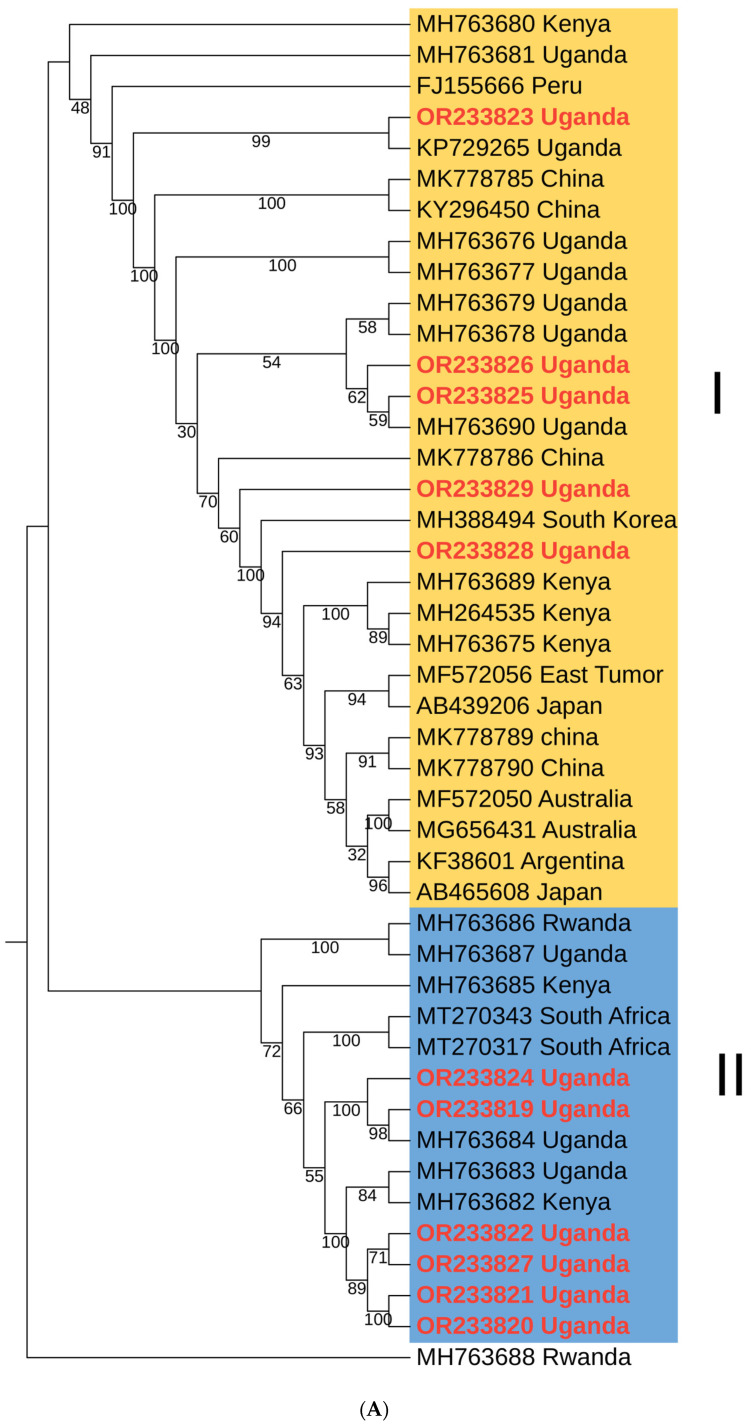

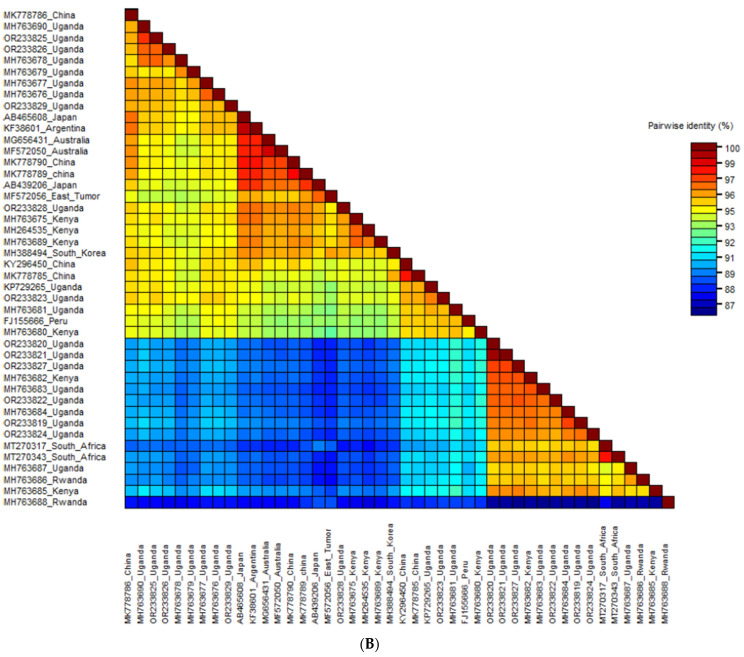
(**A**) Phylogenetic tree of sweet potato feathery mottle virus (SPFMV) constructed using polyprotein-encoding nucleotide sequences and inferred by the maximum likelihood method based on the General Time Reversible model in MEGA11. The numbers at each node indicate bootstrap values. SPFMV_RC MH763668 was used as an out group. (**B**) Color-coded nucleotide identity matrix showing global identity statistics between SPFMV whole genome sequences.

The nucleotide sequences of SPFMV polyproteins were analyzed through phylogenetic methods, revealing the presence of two distinct clusters, labeled as cluster I and cluster II (Figure 4A). Cluster I encompassed sequences from diverse geographical regions, including East Africa (Uganda and Kenya) and countries in Asia, while cluster II mainly consisted of sequences from Africa, particularly East Africa and few sequences from South Africa. This observation highlighted wider geographical distribution of the SPFMV viruses within cluster I group, which occurred in most sweetpotato growing countries of the world, while there was limited geographical distribution of cluster II, which mainly occurred within Africa. The nucleotide identity within cluster II was notably higher than that of cluster I. Additionally, the nucleotide identity between cluster I and cluster II was found to be lower than 93% (Figure 4B).

#### 3.3.3. Phylogenetic Analysis and Nucleotide Sequence Identities of Sweet Potato Virus C

**Figure 5:**
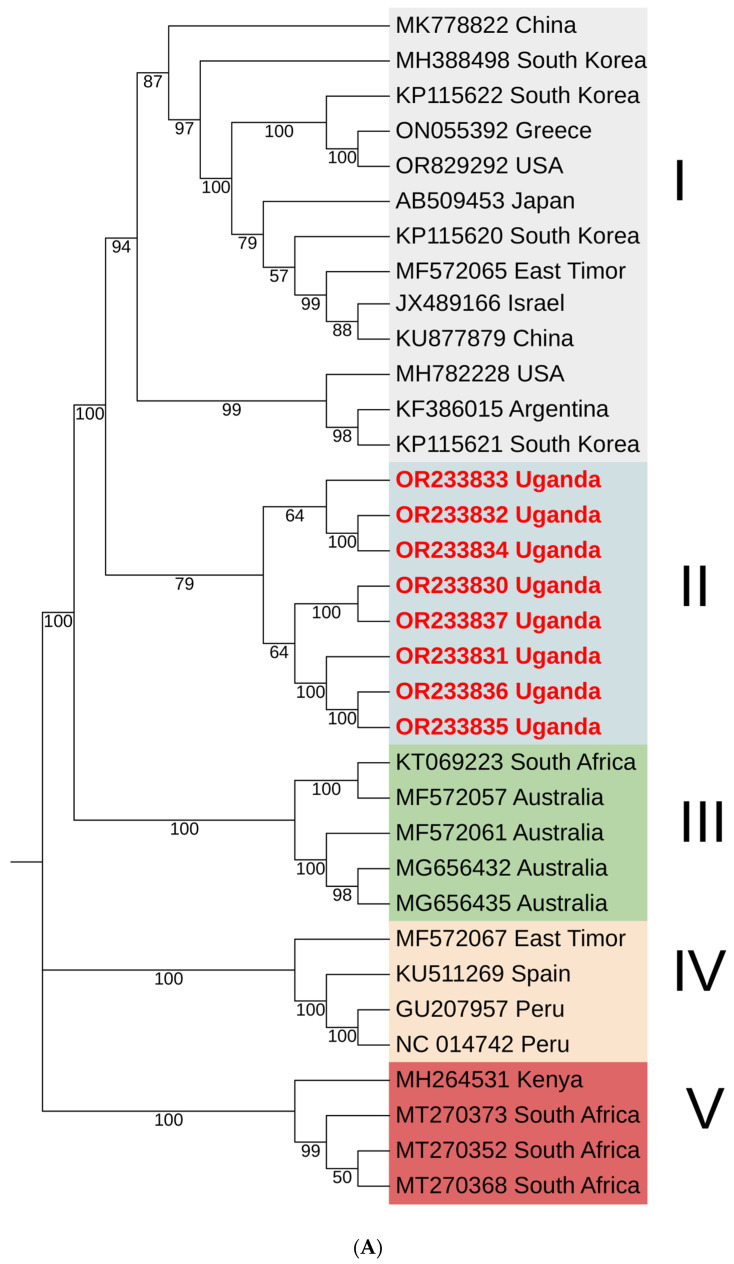

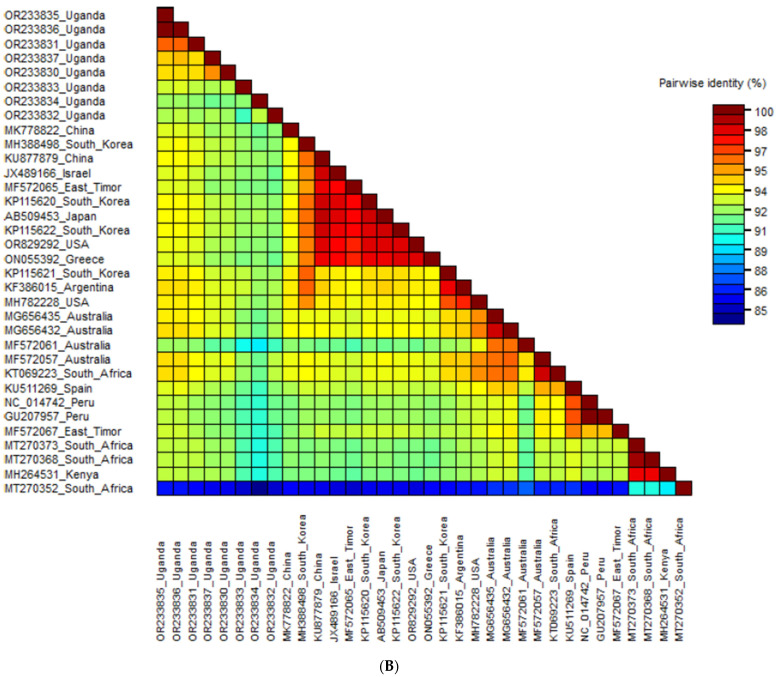
(**A**) Phylogenetic tree of sweet potato virus C (SPVC) constructed using polyprotein encoding nucleotide sequences inferred by the maximum likelihood based on the General Time Reversible model in MEGA11. The numbers at each node indicate bootstrap values. (**B**) Color-coded nucleotide identity matrix showing global identity index statistics between SPVC whole genome sequences.

The phylogenetic analysis of the SPVC polyprotein revealed five distinct phylogroups (I–V). Phylogroup I included sequences from multiple continents, such as Asia, South America, North America and Europe, showing the highest diversity with three sub-clusters and two sequences that did not cluster well with others. SPVC-II comprised sequences exclusively from Uganda in this study, forming two sub-clusters distinct from other sequences. SPVC-III predominantly included sequences from Australia and one sequence from South Africa, further subdivided into two clusters. SPVC-IV consisted of sequences from South America (Peru), Europe (Spain) and East Timor (Asia). Phylogroup SPVC-V exclusively contained sequences from Africa (Kenya and South Africa) (Figure 5A). Within SPVC, nucleotide sequence similarity ranged from 88% to 99% (Figure 5B).

### 3.4. Recombination Analysis of Sweetpotato Potyviruses

Recombination analysis revealed 16 significant recombination events in SPFMV sequences from Uganda, with more events occurring in Phylogroup I than in Phylogroup II. Recombination events were also observed between sequences from Phylogroup I and Phylogroup II. In SPVC sequences from Uganda, 12 recombination events were identified, all occurring in cluster II sequences. Recombination events, similar to those observed in SPFMV, occurred between different phylogroups. Analysis of breakpoint positions indicated that the CP, P1 and CI genes were likely hotspots for recombination in SPFMV. Additionally, HC-Pro was identified as a hotspot in SPVC. No breakpoints were observed in the P3 and 6K1 genes of both potyviruses or in the NIA-Pro gene of SPFMV (Table 2).

### 3.5. Nucleotide Diversity and Neutrality Test

The overall nucleotide diversity score for SPFMV sequences was 0.07661, with the highest diversity observed in sequences from Uganda in group I (0.06190). SPFMV exhibited a total of 3601 mutations, with sequences from Uganda in this group accounting for 2643 mutations. In SPVC sequences, the total nucleotide diversity score was 0.07008. Within SPVC clusters, cluster II, comprising sequences exclusively from Uganda, showed the highest diversity score compared to sequences from other countries, similar to observations in SPFMV. Negative values were observed for Tajima’s D, Fu and Li’s D, and Fu and Li’s F statistics across all phylogroups and clusters for both SPFMV and SPVC, except for cluster IV in SPVC (Table 3).

### 3.6. Selection Pressure of Sweetpotato Potyviruses

Purifying selection was the primary force of selection for all three viruses (SPFMV, SPVC and SPCFV) across all genes (Table 4). Some codons across different genes were found to undergo diversifying selection within the three viruses (Table 4). In SPFMV, the P1 gene exhibited the highest positive diversifying selection pressure. Both FEL and MEME identified a union of 10 codons (253, 263, 267, 429, 449, 473, 533, 575, 576 and 668) within the P1 gene of SPFMV as being under positive diversifying selection. For the P3 gene, codon number 131 was confirmed by both FEL and MEME, while codons 155 and 192 were identified by MEME alone as being under positive diversifying selection. Regarding the CI gene, FEL predicted codon 120 and MEME predicted codons 105 and 511 to be under positive diversifying selection. In the CP gene, FEL predicted codons 2 and 4, while MEME predicted codons 32, 52, 76 and 259 to be under positive selection. Finally, codon 346 in the NIB gene was predicted by FEL and codon 349 was predicted by MEME to be under positive diversifying selection.

In SPVC, the P1 gene had a higher occurrence of positive diversifying selection than was observed in SPFMV. Both FEL and MEME identified a union of six codons (255, 352, 353, 354, 496, 543) within the P1 gene of SPVC as being under positive diversifying selection. In the HC-Pro gene, MEME predicted codons 84, 85 and 236 to be under positive diversifying selection. Codons 131, 155 and 192 in the P3 gene, codons 50 and 370 in the NIB gene, and codon 24 in the CP gene were predicted to undergo diversification. In SPCFV, positive diversifying selection occurred codon 44 in the TGB1 gene, and codons 6 and 8 in the CP gene were identified using both the FEL and MEME methods.

Purifying selection was the predominant force of selection for all three viruses (SPFMV, SPVC and SPCFV) across all genes (Table 4). However, some codons within different genes were found to be undergoing diversifying selection in these viruses (Table 4). In SPFMV, the P1 gene exhibited the highest positive diversifying selection pressure. Both FEL and MEME identified a set of 10 codons (253, 263, 267, 429, 449, 473, 533, 575, 576 and 668) within the P1 gene of SPFMV under positive diversifying selection. For the P3 gene, codon 131 was confirmed by both FEL and MEME, while MEME alone identified codons 155 and 192 under positive diversifying selection. In the CI gene, FEL predicted codon 120 and MEME predicted codons 105 and 511 to be under positive diversifying selection. For the CP gene, FEL predicted codons 2 and 4 and MEME predicted codons 32, 52, 76 and 259 to be under positive selection. Finally, codon 346 in the NIB gene was predicted by FEL and codon 349 was predicted by MEME to be under positive diversifying selection.

In SPVC, the P1 gene showed a higher occurrence of positive diversifying selection compared to SPFMV. Both FEL and MEME identified 6 codons (255, 352, 353, 354, 496, 543) within the P1 gene of SPVC under positive diversifying selection. MEME also predicted codons 84, 85 and 236 in the HC-Pro gene to be under positive diversifying selection. Additionally, codons 131, 155 and 192 in the P3 gene, codons 50 and 370 in the NIB gene, and codon 24 in the CP gene were predicted to undergo diversification. In SPCFV, positive diversifying selection was observed at codon 44 in the TGB1 gene, and codons 6 and 8 in the CP gene were identified using both the FEL and MEME methods.

### 3.7. Genetic Differentiation

The genetic differentiation observed in SPFMV sequences was higher compared to SPVC sequences encoding the polyprotein. We identified significant genetic differences between cluster I and cluster II of SPFMV, with an Fst value of 0.57615, indicating substantial genetic disparities between these two groups. Moreover, within Uganda, we observed greater differentiation between sequences in cluster I and cluster II (Table 5). Conversely, in the case of SPVC, the lowest genetic differentiation was observed between the Uganda variant (cluster II) and all other clusters. However, genetic differentiation above 0.4 was evident among the other clusters (Table 5).

## 4. Discussion

This study presents de novo whole genome sequences of SPFMV, SPVC and SPCFV from Uganda, including the first de novo whole genome sequences of sweet potato feathery mottle strain O.

Our study also reports the first report of SPSMV_1 and SPPV in Uganda (Supplementary Table S3). These viruses have been previously detected in other countries such as Tanzania, Central American countries, Korea, China, Europe and South Africa [48,49,50,51,52]. Their introduction to Uganda may be attributed to cross-continental spread, which often occurs inadvertently through the trade or exchange of infected plant materials between countries. This process can transfer hosts, viruses and vectors to new regions and environments [53]. Additionally, SPSMV_1 and SPPV might have gone undetected in Uganda by commonly used detection methods, such as antibody- and nucleic acid-based techniques, which require prior knowledge of the virus in question [22,25]. Further studies are needed to quantify yield losses associated with the co-infection of newly detected viruses with other viruses.

Our study reported an increased occurrence of SPFMV_O in Uganda, contrasting earlier studies that indicated a low prevalence within East Africa [13,20]. This suggests that SPFMV_O is becoming increasingly significant in sweet potato viral infections in Uganda. Modern agricultural practices, such as monoculture, extended growing seasons, irrigation and artificial soil amendments, have influenced plant pathogen prevalence, including viruses [20,53]. In this study, SPCFV was found to play a role in co-infections, but only in combination with SPFMV, SPVC and SPCSV. Although the vector for SPCFV is unknown, Tugume et al. [21] observed that SPCFV and SPFMV might be transmitted by a common vector due to the frequent co-infection of these viruses.

Whole genome sequences of SPCFV obtained in this study were typical of *Carlavirus* [54]. The sequence identity and similarity of SPCFV sequences from East Africa were high compared to sequences from other geographic regions. Phylogenetic tree analysis revealed three distinct genetic lineages, clustering sequences into phylogroups well associated with geographic location. This suggests closer genetic connectivity within SPCFV sequences from East Africa, indicating a probable common origin or descent of the virus and confirming a higher genetic diversity in the sequences from outside East Africa. The whole genome sequences of SPFMV and SPVC obtained in our study were typical of the family *Potyviridae* [55,56,57]. Phylogenetic analysis of the polyprotein of SPFMV sequences revealed two major clusters (I and II). Cluster I contained sequences from both SPFMV strains O and EA, while cluster II contained sequences from SPFMV strain EA only. In contrast, phylogenetic analysis of the polyprotein of SPVC sequences showed five sub-clusters, indicating higher diversification in SPVC compared to SPFMV. Notably, SPVC sequences from Uganda did not cluster with sequences from any other regions, including East Africa, suggesting that SPVC in Uganda is potentially evolving independently from SPVC in other countries.

Comparable findings of higher genetic diversity scores among SPFMV and SPVC sequences from Uganda compared to sequences from other countries confirm previous reports showing a high rate of evolution of plant viruses in East Africa [13,16,20]. East Africa has been considered a hotspot for the diversification of major plant viruses infecting economically important crops, like sweet potato, cassava and rice [21]. Strains of sweetpotato viruses, such as SPCSV, SPFMV, SPMMV and the proposed SPCFV strain East Africa [13,21], were first reported and restricted to East Africa until recently. The high genetic diversity of plant viruses detected in Uganda and East Africa at large results from several interconnected factors. These include diverse geography that creates different ecological niches [58], environmental heterogeneity with variations in temperature, humidity and rainfall patterns that influence the survival, spread and mutation rates of plant viruses [59] and host plant diversity, as Uganda is rich in crop cultivars that can host different viral strains, thereby promoting genetic diversity [60]. Additionally, the practice of intercropping sweetpotatoes with other crops creates opportunities for cross-infection and the emergence of new viral strains [61]. Vector dynamics, characterized by a diversity of vector species and their behaviors, influence virus transmission and genetic mixing [62]. Human agricultural practices, such as the movement of planting materials [63] and seed exchange networks [64], also play a significant role alongside intrinsic evolutionary processes, like high mutation and recombination rates [65]. The higher diversity scores in SPFMV and SPVC sequences from Uganda compared to sequences from other geographical locations suggest lower genetic stability of both viruses, calling for constant monitoring and further studies to understand the evolutionary forces behind this pattern.

Plant viruses naturally generate genetic diversity through mutation, reassortment and recombination [66]. In this study, a high number of mutations were observed in SPFMV and SPVC. SPFMV and SPVC are single-stranded RNA viruses that have higher mutation rates than DNA viruses because RNA polymerases lack proofreading activity. As a result, any mismatches are not corrected, leading to genome replication errors due to the misincorporation of nucleotides in the daughter strand and thus resulting in high mutation rates [67].

In addition to mutation, another critical source of genetic variation in both DNA and RNA viruses is genetic exchange, which can occur through recombination or reassortment. Recombination was not detected among SPCFV sequences, implying that other forces are driving the evolution of SPCFV, necessitating further investigation to identify these forces. Recombination is an event coupled with virus replication and is facilitated by mixed infections of two or more viruses [68]. During mixed infection, potyviruses can recombine their genetic material, leading to intra- and inter-strain recombination.

Recombination has been implicated in misrepresenting the true phylogenetic relationships of viruses [65,66,68,69]. In our study, phylogenetic analysis of SPFMV sequences clustered SPFMV strain O and SPFMV strain EA together into cluster I, which included recombinant sequences identified in our study. It is important to note that recombinant sequences were included in the phylogenetic analysis because ignoring recombination can lead to a large overestimation of the substitution rate heterogeneity and the loss of the molecular clock [68].

Recombination does not occur randomly across the genome. Instead, it tends to occur preferentially at “hotspots” within a virus genome. In this study, recombination hotspots identified for SPFMV and SPVC include the nuclear inclusion protein a-proteinase (Nia-pro), nuclear inclusion protein b (NIb), coat protein (CP), protein 1 (P1) and helper component proteinase (HC-PRO) regions, as previously reported [70,71,72,73,74]. Identifying hotspots in virus genomes is fundamental to understanding the role of recombination in evolution, as these hotspots may signify areas of the genome that are mutationally robust and do not have deleterious effects on the fitness of the novel progeny [66].

The CP gene was the most susceptible to recombination. This gene is crucial in the viral life cycle and host plant defense mechanisms and has also been widely utilized in virus diagnosis, studying viral populations, genetic diversity and conducting phylogenetic analysis across many plant viruses. This susceptibility poses significant challenges for host responses because the CP gene is integral to virus survival and host defense. Additionally, since the CP gene is a primary target for virus diagnostics, any genetic divergence in this region can complicate the accurate identification of virus species or strains [75].

Recombination has driven the divergence of numerous viruses, leading to the emergence of new, more virulent strains. The consequences of recombinant viruses have been evident in various diseases and epidemics, primarily because they can overcome resistance mechanisms [21,76,77,78,79,80,81,82]. An example is the East African cassava mosaic virus-Ugandan strain (EACMV_UG), a recombinant between two cassava mosaic geminiviruses. This strain increased symptom severity and caused severe epidemics of cassava mosaic disease, leading to significant food loss and shortages across Uganda in the 1990s and has since extended to neighboring countries [8].

This highlights that recombination events can significantly alter the viral population structure. Because recombination has been associated with the emergence of viruses and increased infectivity observed in various crops leading to novel virus epidemics [76], it should not be overlooked as a significant evolutionary process [66]. Recombination has also facilitated cross-species transmission to wild hosts. In sweetpotato, viruses such as SPCFV, SPCSV, SPFMV, SPVC and SPMMV were detected in wild Convolvulaceae plants in Uganda, suggesting a bidirectional movement of SPCFV between wild and cultivated plants [13,21]. 

Recombination plays a critical role in the evolution of viruses, contributing significantly to genetic diversity and the emergence of new epidemics in crops [76,77,78,79,80,81,82]. It is a key process in viral adaptability and pathogenicity, influencing variations in virulence and the geographical origin of virus strains. Understanding this process is vital for designing effective disease control strategies, particularly in breeding programs aimed at developing resistant plant varieties. However, the genetic variability of viruses is often overlooked by plant breeders in favor of host genetic variability in plant breeding efforts, which may hinder comprehensive disease management [83].

Diverse virus populations can facilitate the evasion of host resistance mechanisms or maintain variants with selective advantages in different environments [84]. Genetic variation resulting from mutation and recombination is shaped by additional evolutionary forces such as natural selection, genetic drift and gene flow. Neutrality tests revealed negative Tajima’s D and F values across SPVC, SPFMV and SPCFV, except for SPVC-IV, indicating deviations from neutral evolution. This negative neutrality suggests a reduction in the frequency of polymorphisms, indicative of population size expansion following purifying selection [85]. These findings were corroborated by FEL and MEME analyses, which underscored the predominant role of purifying selection over positive diversifying selection across all genes in SPFMV, SPVC and SPCFV. Selection can either be positive (diversifying), increasing the frequency of beneficial alleles, or negative (purifying), decreasing the frequency of harmful alleles and responding to changes in environmental conditions [86]. Purifying selection acts to reduce genetic diversity, slow the evolutionary rate, and minimize changes to amino acids, thereby safeguarding essential viral functions against deleterious mutations. This emphasizes the critical role of purifying selection in maintaining the genetic stability of sweetpotato potyviruses and offers valuable insights into the evolutionary mechanisms shaping their genome [85,86].

While negative selection predominated in our study, positive diversifying selection was observed in certain codons across the genomes, with the highest occurrences in SPVC, followed by SPFMV. Detection of positive selection signatures in a genome signifies adaptation in response to environmental changes during population evolution [87].

Specifically, the P1 gene exhibited the highest number of codons under positive selection in both potyviruses, consistent with the findings by Wokorach et al. 2020 [20]. The P1 gene is pivotal in virus infectivity, RNA replication, suppression of RNA interference (RNAi) and potentially in host adaption. Specifically, the P1 gene exhibited the highest number of codons under positive selection in both potyviruses, consistent with findings. The P1 gene is pivotal in virus infectivity, RNA replication, suppression of RNA interference (RNAi) and potentially in host adaptation [55].

Selection pressures from vectors, hosts and environmental factors drive the extinction of less-adapted variants, facilitating the emergence of new viral strains or species with altered biological properties, such as widened host ranges, resistance-breaking capabilities, or novel disease symptoms. These changes can lead to epidemics that pose significant threats to global food security [74]. SPFMV exhibited higher genetic differentiation compared to SPVC, particularly in sequences from Uganda, relative to those from other countries. Conversely, SPVC sequences from Uganda displayed lower genetic differentiation compared to SPVC from elsewhere. High genetic differentiation suggests limited gene flow between populations due to independent evolutionary processes. The greater gene flow observed in SPVC compared to SPFMV within the same study area likely results from a combination of factors including viral genetic diversity, adaptability to hosts and vectors, vector efficiency, interactions between plants and viruses, agricultural practices (such as cultivation patterns and human-mediated movement, and environmental conditions and genetic stability). Understanding these factors is crucial for developing targeted strategies to manage virus spread in sweetpotato crops.

## 5. Conclusions

This study marks the first detection of SPPV and SPSMV_1 in Uganda, revealing their presence in local sweetpotato varieties and emphasizing the urgency of implementing surveillance and management strategies to mitigate potential impacts on sweetpotato production. This initial detection represents a critical step towards mapping the distribution of these viruses and guiding future agricultural practices to safeguard essential crops in Uganda. This study identified mutations, significant recombination events and positive diversifying selection in the P1 regions, along with purifying selection across the SPFMV and SPVC genomes, all of which play pivotal roles in shaping the genetic diversity and evolution of sweetpotato viruses. These findings suggest the potential emergence of new variants of sweetpotato virus strains that could pose new disease challenges and complicate current control strategies in Uganda and the region, potentially affecting accurate diagnostic identification for disease management. The newly published whole-length genome sequences of SPFMV, SPVC and SPCFV will serve as valuable resources for future molecular studies aimed at further elucidating virus evolution, dispersal and virulence. Therefore, the information generated in this study is instrumental for sweetpotato breeding programs in the region, informing strategies for managing virus-related diseases, enhancing virus diagnostics and breeding resistant varieties to support resource-poor farmers who rely heavily on sweetpotato for food security, income and nutritional needs.

## Figures and Tables

**Table 1:** Sweet potato viruses detected in regions surveyed in Uganda.

Region	District	Field Number	SPFMV_EA	SPFMV_O	SPVC	SPCSV	SPMMV	SPCFV	SPLCV	SPPV	SPSMV_1	SPCV
Central	Wakiso	WAK3	1	1	1	1	0	0	0	0	0	0
		WAK2	1	1	0	0	0	0	1	0	0	0
		WAK4	1	0	1	1	0	0	1	0	0	0
		NaCRRI	1	0	1	0	0	0	0	1	0	0
	Mukono	MKN 4	1	0	0	0	0	0	0	0	0	0
		MKN 5	1	1	1	1	0	0	0	0	0	0
		MKN 3	1	1	1	1	0	0	1	1	0	0
	Mpigi	MPG 5	1	0	1	1	0	1	1	1	0	0
		MPG 6	1	1	1	1	0	0	1	1	0	0
		MPG 2	1	0	1	1	0	1	0	0	0	0
	Luwero	LUW 9	1	0	1	1	0	0	1	1	0	0
		LUW 4	1	0	0	1	0	0	0	0	0	0
Eastern	Iganga	IGA 5	1	1	1	1	0	1	0	1	0	0
		IGA 4	1	1	0	0	0	0	1	0	0	0
		IGA 1	1	0	1	0	0	0	0	0	0	0
	Serere	SER 4	1	0	1	1	0	0	1	0	0	0
		SER 2	1	0	1	1	0	0	0	0	0	0
	Soroti	SRT 3	1	0	0	1	0	0	0	0	0	0
		SRT 5	1	0	1	1	0	0	0	0	0	0
		SRT 4	0	0	0	1	0	0	1	0	0	0
	Kumi	KMI 3	1	0	1	1	0	0	0	1	0	0
		KMI 4	1	0	1	1	0	0	0	1	0	0
		KMI 5	1	1	1	0	0	0	0	0	0	0
	Kamuli	KML 4	1	0	1	1	0	0	1	0	0	0
		KML 3	1	1	0	1	0	0	1	0	0	0
		KML 5	1	0	1	1	0	1	0	1	0	0
	Mbale	MLE 1	1	0	1	1	1	1	0	0	0	0
		MLE 4	1	1	1	1	0	0	0	1	0	0
Western	Bushenyi	BSH 5	1	0	1	0	0	0	0	1	0	0
		BSH 6	1	0	1	1	0	1	0	1	0	0
	Kabarole	KAB 2	1	0	0	0	0	0	0	0	0	0
		KAB 5	1	1	0	0	0	0	0	0	0	0
	Kasese	KAS 3	1	1	1	0	0	0	0	0	0	0
	Mubende	MUB 1	1	0	0	0	0	0	0	0	0	0
		MUB 3	1	1	1	1	0	0	0	1	0	1
	Hoima	HMA 7	1	0	1	0	0	0	0	1	1	0
		HMA 4	1	0	1	1	0	0	0	0	0	0
Control	KEPHIS	Control	0	0	0	0	0	0	0	0	0	0
Total detection rate (%)			94.7	34.2	71.1	65.8	2.6	15.8	28.9	36.8	2.6	2.6

Viruses detected include: SPFMV_EA (sweet potato feathery mottle virus_East African strain), SPFMV_O (sweet potato feathery mottle virus_ordinary strain), SPVC (sweetpotato virus C), SPCSV_EA (sweet potato chlorotic virus_East African strain), SPMMV (sweet potato mild mottle virus), SPCFV (sweet potato chlorotic fleck virus), SPLCV (sweet potato leaf curl virus), SPPV (sweet potato pakakuy virus), SPSMV_1 (sweet potato symptomless virus_1), SPCV (sweet potato collusive virus), control (Sample from Kenya Plant Health Inspectorate Services (KEPHIS)); Districts in Uganda: WAK (Wakiso), MKN (Mukono), MPG (Mpigi), LUW (Luweero), IGA (Iganga), SER (Serere), SRT (Soroti), KMI (Kumi), KML (Kamuli), MLE (Mbale), BSH (Bushenyi), KAB (Kabarole), KAS (Kasese), MUB (Mubende), HMA (Hoima).

**Table 2:** Predicted recombination events and recombinant sequences of SPFMV and SPVC.

Breakpoint Position in Recombinant			Detection Methods							
Begin	End	Recombinant Sequence	Minor Parent	Major Parent	RDP	GENECOV	BootScan	MaxChi	Chimaera	SiSscan
SPFMV										
2963	10,479	^ MH763690	MH763678	OR233827	2.76 × 10^−24^	4.05 × 10^−37^	1.41 × 10^−34^	3.45 × 10^−13^	4.67 × 10^−11^	7.42 × 10^−77^
3347	6656	^ MH763680	MK778789	OR233827	1.45 × 10^−80^	1.17 × 10^−77^	6.37 × 10^−77^	4.80 x10−44	5.13 × 10^−44^	4.45 × 10^−58^
7597	10,291	^ KP729265	MH763687	AB465608	7.47 × 10^−75^	2.72 × 10^−43^	7.61× 10^−73^	3.31 × 10^−31^	8.95 × 10^−34^	1.92 × 10^−29^
3473	6829	^ MH763681	MK778789	MH763685	1.32 × 10^−70^	1.78 × 10^−65^	1.62 × 10^−68^	1.46 × 10^−40^	5.92 × 10^−11^	1.56 × 10^−52^
8409	9995	MH763677	MH763686	AB465608	9.76 × 10^−67^	4.95 x10−45	2.32 × 10^−66^	8.83 × 10^−21^	4.57 × 10^−22^	1.57 × 10^−23^
3168	8405	OR233825	AB439206	^ OR233819	1.83 × 10^−17^	3.46 × 10^−16^	NS	4.28 × 10^−14^	1.75 × 10^−15^	NS
6827	7304	^ MH763687	* MH388494	OR233821	NS	6.86 × 10^−24^	2.12 × 10^−33^	3.47 × 10^−09^	8.87 × 10^−12^	NS
6816	7314	^ MH763687	MH763676	OR233819	2.48 × 10^−34^	5.01 × 10^−24^	NS	1.85 × 10^−10^	1.99 × 10^−13^	NS
8421	10,000	OR233825	^* MH763675	FJ155666	1.43 × 10^−13^	1.90 × 10^−09^	NS	7.76× 10^−14^	1.98 × 10^−17^	NS
10,476	813	MH763678	* OR23382	MH763687	6.22 × 10^−12^	2.67 × 10^−24^	3.08 × 10^−24^	5.90 × 10^−16^	8.82 × 10^−11^	9.71 × 10^−23^
8046	10,489	^ MH763680	MH763683	MG656431	4.65 × 10^−04^	NS	3.97 × 10^−08^	8.67 × 10^−11^	1.14 × 10^−08^	1.68 × 10^−24^
8406	10,268	MH763687	* MH763683	^ MK778789	3.31× 10^−04^	5.47 × 10^−05^	5.15 × 10^−06^	9.94 × 10^−09^	5.31 × 10^−06^	9.96 × 10^−20^
822	1185	MH763677	* MK778790	^ FJ155666	9.64 × 10^−05^	1.48 × 10^−08^	5.60 × 10^−05^	1.07 × 10^−05^	6.94 × 10^−03^	8.80 × 10^−11^
1637	2962	MH763690	OR233825	MH763685	1.09 × 10^−04^	NS	1.09 × 10^−05^	2.22 × 10^−03^	1.04 × 10^−06^	2.83 × 10^−05^
625	921	^ MH763675	* MH763676	MH763683	1.19 × 10^−04^	NS	5.16× 10^−05^	1.91 × 10^−03^	4.83× 10^−04^	1.25 × 10^−04^
10,007	10,273	^ OR233824	* MH763688	OR233822	4.61 × 10^−24^	NS	3.79 × 10^−02^	2.06 × 10^−03^	3.76 × 10^−02^	NS
SPVC										
10,409	1152	^ OR233832	* MT270352	MT270368	6.12 × 10^−68^	2.76 × 10^−65^	1.21 × 10^−65^	6.56 × 10^−27^	2.86 × 10^−26^	1.24 × 10^−38^
7341	9181	^ OR233834	KU877879	OR233833	2.06 × 10^−22^	2.12 × 10^−24^	9.85 × 10^−26^	4.77 × 10^−15^	5.21 × 10^−18^	1.14 × 10^−24^
6669	8551	^ OR233833	OR233832	OR233835	5.24 × 10^−23^	5.65 × 10^−19^	8.95 × 10^−23^	3.21 × 10^−17^	1.27 × 10^−16^	7.71 × 10^−26^
2271	6668 *	OR233834	OR233833	* MF572067	9.88 × 10^−05^	NS	2.32 × 10^−06^	1.44 × 10^−11^	6.76 × 10^−08^	3.42 × 10^−25^
6751	9910	^ OR233832	* KF386015	MH782228	1.43 × 10^−06^	1.02 × 10^−15^	5.06 × 10^−16^	1.39 × 10^−14^	3.34 × 10^−10^	6.91 × 10^−35^
5515	10,391	^ OR233831	* OR233837	OR233835	NS	NS	NS	2.08 × 10^−13^	9.39 × 10^−13^	NS
1722	6344 *	^ OR233833	* MT270373	MH264531	NS	NS	1.84 × 10^−05^	0.00367815	0.03060258	3.41 × 10^−45^
10,305	1710 *	^ OR233837	* KF386015	OR233835	0.01606069	NS	NS	1.49 × 10^−09^	4.44 × 10^−08^	NS
9935	6344 *	^ OR233835	KF386015	MT270368	NS	NS	NS	1.57 × 10^−06^	NS	1.82 × 10^−09^
1167 *	1647	OR233832	OR233834	MF572057	NS	NS	0.00440029	8.59 × 10^−06^	1.58 × 10^−07^	0.002772
9270 *	10,008 *	^ OR233834	ON055392	* KP115621	0.00106145	NS	NS	0.00347041	0.00024433	8.06 × 10^−08^
8861 *	10,275	^ OR233833	OR829292	KF386015	NS	NS	NS	0.00748995	0.00046536	NS

^ = The recombinant sequence may have been misidentified (one of the identified parents might be the recombinant), * = Unknown parent. The reported *p*-value is the highest *p*-value among those calculated using RDP4-implemented methods, where NS = No significant *p*-value was recorded for this recombination event using this method.

**Table 3:** Nucleotide diversity calculated from polyprotein nucleotide sequences of SPFMV and SPVC.

	Population	N	S	M	Pi	K	D	Fu and Li’s D Statistics	Fu and Li’s F Statistics	(*p*-Values)
SPFMV	Group I	31	2903	3601	0.05694	523.31773	−1.33133	−0.99723	−1.31807	NS, *p* > 0.10
	Group II	14	1832	1917	0.03960	415.08791	−1.41274	−1.24259	−1.48322	NS, *p* > 0.10
	Uganda (group I)	12	2459	2643	0.06190	653.49451	−0.96972	−0.96972	−0.82041	NS, *p* > 0.10
	Uganda (group II)	9	1326	940	0.03669	387.361	−1.17733	−1.12643	−1.28085	NS, *p* > 0.10
	Total estimates	44	3746	4410	0.07661	798.69556	−0.78956	−1.12583	−1.19627	NS, *p* > 0.10
SPVC	Cluster I	14	1542	1786	0.04299	449.07692	−0.90899	−1.11134	−1.21390	NS, *p* > 0.10
	Cluster II (Uganda Variant)	8	1858	1963	0.06448	671.07143	−0.62518	−0.47712	−0.57216	NS, *p* > 0.10
	Cluster III	5	807	822	0.03653	381.60000	−0.25120	−0.20468	−0.23347	NS, *p* > 0.10
	Cluster IV	4	633	641	0.03397	354.83333	0.15619	0.03416	0.20403	NS, *p* > 0.10
	Cluster V	4	1146	1162	0.05680	586.50000	−0.78478	−0.74056	−0.80874	NS, *p* > 0.10
	Total estimates	34	3588	4220	0.07008	723.55793	−1.15053	−1.18110	1.39161	NS, *p* > 0.10

N = number of sequences, S = No. of segregating site, M = Number of mutations, Pi = Nucleotide diversity, K = Average number of differences, D = Tajima’s neutrality test, Fu and Li’s D statistics, Fu and Li’s F statistics, NS = Non-significant.

**Table 4:** Number of codons under selection in the genomic regions of SPFMV, SPVC and SPCFV.

Codon Sites Predicted Undergoing Positive Diversifying Selection				No. of Codons Undergoing Purifying Selection
Virus	Gene	FEL	MEME	FEL
SPFMV	P1	**253, 263, 267**, 290, **429**, **449**, **473**, **533, 575**, **576**, **668**	8, 69, 89, **253**, **263**, **267**, 271, 275, 414, **429**, **449**, **473**, **533**, **575**, **576**, **668**	177
	HC-Pro	-	3, 9	202
	P3	**131**	**131**, 155, 192	131
	6K1	-	-	28
	CI	120	105, 511	423
	6K2	-	-	24
	NIA-VPG	-	-	89
	NIA-PRO	-	98	123
	NIB	346	349	239
	CP	2, 4	32, 52, 76, 259	122
SPVC	P1	**255**, **352**, **353**, **354, 496**, **543**, 602, 613	35, 37, 40, 43, 45, 108, 172, 202, 235, **255,** 267, 280, 349, **352**, **353**, **354**, 406, 410, 465, 487, **496**, 497, 503, **543**, 598, 634	111
	HC-Pro	**85**	84, **85,** 236	156
	P3	**173**	161, **173**, 308	86
	6K1	-	-	7
	CI	-	196, 270, 293	280
	6K2	-	-	13
	NIA-VPG	-	-	64
	NIA-PRO	-	-	97
	NIB	**50**	**50**, 370	243
	CP	**24**	**24**	89
SPCFV	TBG1	**44**	**44**	109
	TBG2	-	-	56
	TBG2	-	-	22
	NaBP	-	-	38
	CP	**6**, **8**	**6, 8**	176

Bold indicate sites predicted by two models to be under positive selection.

**Table 5:** Genetic differentiation between the different phylogroups of SPFMV and SPVC based on polyprotein sequences.

Viruses	Population 1	Population 2	Gst	DeltaSt	GammaSt	Nst	Fst
SPFMV	Group I	Group II	0.00321	0.02777	0.38359	0.59408	0.57615
	Uganda variant (Group I)	Uganda variant (group II)	0.00015	0.03109	0.45252	0.59811	0.58139
SPVC	Cluster II (Uganda variant)	Cluster I	0.00285	0.0115	0.20538	0.26884	0.26386
	Cluster II (Uganda variant)	Cluster III	0.00431	0.0136	0.22782	0.30058	0.29366
	Cluster II (Uganda variant)	Cluster IV	0.04729	0.01723	0.27107	0.38994	0.381
	Cluster II (Uganda variant)	Cluster V	0.01031	0.02114	0.289	0.38734	0.37452
	Cluster I	Cluster III	0.01349	0.01282	0.2691	0.42023	0.41169
	Cluster I	Cluster IV	0.05134	0.01522	0.30718	0.50541	0.49565
	Cluster I	Cluster V	0.02239	0.02043	0.34772	0.50996	0.49837
	Cluster III	Cluster IV	0.04382	0.01769	0.3909	0.45062	0.44172
	Cluster III	Cluster V	0.00139	0.02597	0.42515	0.48303	0.47312
	Cluster IV	Cluster V	0.04348	0.02691	0.44103	0.49272	0.4828

## Data Availability

Whole genome sequences from this study have been deposited into the NCBI GenBank database under accession numbers OR233819–OR233837 and OR233992–OR233994.

## Supplementary Materials

The following supporting information can be downloaded at: https://www.mdpi.com/article/10.3390/pathogens13100833/s1, Table S1: Occurrence of SPVD-like symptoms in sweetpotato in districts surveyed; Table S2: Number of reads generated from sequencing, before and after quality trimming; Table S3: Sweetpotato viruses detected in 38 samples sequenced from 16 districts in Uganda; Table S4: Reference genome sequences from different regions worldwide were obtained from NCBI GenBank used in this study.
